# Genomic Scans of Zygotic Disequilibrium and Epistatic SNPs in HapMap Phase III Populations

**DOI:** 10.1371/journal.pone.0131039

**Published:** 2015-06-30

**Authors:** Xin-Sheng Hu, Yang Hu

**Affiliations:** 1 Department of Plant Sciences, University of Oxford, South Parks Road, Oxford, OX13RB, United Kingdom; 2 Department of Computing Science, University of Alberta, Edmonton, AB T6G 2S4, Canada; Pennsylvania State University, UNITED STATES

## Abstract

Previous theory indicates that zygotic linkage disequilibrium (LD) is more informative than gametic or composite digenic LD in revealing natural population history. Further, the difference between the composite digenic and maximum zygotic LDs can be used to detect epistatic selection for fitness. Here we corroborate the theory by investigating genome-wide zygotic LDs in HapMap phase III human populations. Results show that non-Africa populations have much more significant zygotic LDs than do Africa populations. Africa populations (ASW, LWK, MKK, and YRI) possess more significant zygotic LDs for the double-homozygotes (*D_AABB_*) than any other significant zygotic LDs (*D_AABb_*, *D_AaBB_*, and *D_AaBb_*), while non-Africa populations generally have more significant *D_AaBb_*’s than any other significant zygotic LDs (*D_AABB_*, *D_AABb_*, and *D_AaBB_*). Average r-squares for any significant zygotic LDs increase generally in an order of populations YRI, MKK, CEU, CHB, LWK, JPT, CHD, TSI, GIH, ASW, and MEX. Average r-squares are greater for *D_AABB_* and *D_AaBb_* than for *D_AaBB_* and *D_AABb_* in each population. YRI and MKK can be separated from LWK and ASW in terms of the pattern of average r-squares. All population divergences in zygotic LDs can be interpreted with the model of Out of Africa for modern human origins. We have also detected 19735-95921 SNP pairs exhibiting strong signals of epistatic selection in different populations. Gene-gene interactions for some epistatic SNP pairs are evident from empirical findings, but many more epistatic SNP pairs await evidence. Common epistatic SNP pairs rarely exist among all populations, but exist in distinct regions (Africa, Europe, and East Asia), which helps to understand geographical genomic medicine.

## Introduction

Epistasis between loci for fitness is one of genetic mechanisms for multilocus evolution in evolutionary biology [1, 2, 3]. For instance, epistatic selection enhances the formation of rugged fitness landscape and hence impedes the shifting of a local population from one fitness peak to another [4, 5, 6]. Epistasis also provides a genetic basis for developing therapeutic strategy in pharmacogenomics [7,8]. In a life cycle of human, many plants and animals, it might occur in either the haploid gametic or diploid zygotic stage, or in both. In terms of quantitative genetics [9], two syntenic or non-syntenic non-alleles in the gametic stage could interact via additive by additive effects on gamete fitness and subsequently reinforce gametic selection, such as the incompatibility of specific non-alleles [10]. The parental and recombinant gametes could have differential strengths of epistatic selection due to distinct allele interactions. One potential outcome from epistatic selection in the gametic stage is the gametic linkage disequilibrium (LD) between two loci at the population level, which measures the association of haplotypes descended from ancestral chromosomes. In the diploid zygotic stage, epistatic selection (either synergistic or antagonistic effects) in a two-locus system could arise from the additive by additive effects between double homozygotes, the additive by dominance effects between the homozygote at one locus and the heterozygote at the other locus, and the dominance by dominance between double heterozygotes. Similarly, one potential outcome from epistatic selection in the zygotic stage is the zygotic LD between loci, which measures the association of diplotypes (the combination of two haplotypes in one individual) descended from ancestral chromosome pairs. Analogous to the concept of gametic LD (the covariance between non-allele frequencies), zygotic LD is termed as the covariance between two-locus genotypic frequencies [11,12, 13, 14]. These two levels of LDs are genetically related, but gametic LD contains the compounded information of epistatic selection from both stages. Zygotic LD is mainly associated with the epistasis in the diploid zygotic stage [15].

The evolutionary processes other than the epistatic selection can also produce both zygotic and gametic LDs, including mating system, migration, and genetic drift. The difference is that these processes mainly generate statistical non-random associations between loci, different from the effects of epistatic selection that generates functional non-random associations. Although zygotic LD has long been conceptually appreciated [11, 12], its statistical properties and evolutionary theory have not been extensively developed [13, 14, 15]. Practical analysis of zygotic LDs is mainly in crops for detecting selection [16, 17], in dogs [18] and cattle [19, 20] for characterizing population structure, and in a mouse hybrid zone for assessing postzygotic isolation [21]. The crucial difference between gametic and zygotic LDs lies in that zygotic LD calculation uses unphased genotypic data, different from gametic LD calculation that uses phased genotypic data. Recently, Hu [15] has showed that zygotic LDs, owning to their diverse patterns under distinct evolutionary processes among two-locus genotypes, are more informative than the commonly used gametic LD in revealing natural population history. In *Homo sapiens* species, gametic LD has been extensively investigated in many populations of different origins, and its pattern is applied for different purposes, such as mapping single nucleotide polymorphisms (SNPs) for causal diseases and examining population demography [22, 23]. However, zygotic LDs have not been investigated [24], and are rarely applied to characterizing human population history. Thus, it is of evolutionary significance to examine the effectiveness of zygotic LD in revealing human population history.

Besides the utility of zygotic LDs for characterizing population history, Hu [15] and Hu and Yeh [21] further show that gametic and composite digenic LDs (low-order LD) are always greater than the maximum zygotic LD (high-order LD) in a population under the processes of migration, genetic drift, and additive selection. Only epistatic selection can produce a contrasting pattern, i.e. the maximum zygotic LD is greater than the gametic or composite digenic LD. Such a difference can be used to detect epistatic selection under a complex background from various evolutionary processes (migration/admixture, drift, and additive selection). The composite digenic LD differs from gametic LD in that it includes the non-random associations between non-alleles from both within gametes and between gametes within individuals [25]. It recovers gametic LD in Hardy-Weinberg equilibrium (HWE) [25]. The composite digenic LD is used to infer Dobzhansky-Muller incompatibility model in a natural mouse hybrid zone [26]. Here, we search for epistatic SNPs by testing the difference between the composite digenic and maximum zygotic LDs in human populations [15, 21].

The new approach differs from the existing methods in searching for epistasis in two aspects. One is that the trait refers to the general fitness, not a specific complex disease that may affect fitness. The epistatic selection for fitness naturally occurs in a population [15, 21], emphasizing the functional interaction between loci. The existing methods for detecting epistasis often work on specific traits, such as the approach of genome-wide association studies (GWAS) for specific complex diseases [27]. The second aspect is that the new method is zygotic LD-based, and epistatic selection is detected on the basis of individual SNP pairs rather than multiple SNP pairs simultaneously. This minimizes the problem of small sample sizes (*n*) with a large number of SNPs (*p*), i.e. *n*<<*p*, which is frequently encountered in detecting multiple epistasis. The existing methods that deal with multiple SNPs under the situation of *n*<<*p*, often require expensive computations or the assumptions of prior parameter distributions [5, 10, 28, 29]. Thus, the zygotic LD-based method provides an addition to the existing methods in searching for genome-wide epistasis arising from genotypic rather than allelic interactions.

Evidence supporting genotypic interactions is recorded in the literature although few studies link the genotypic interaction with zygotic LDs at the population level [21]. For instance, the double heterozygotes (one locus for insulin receptor and the other locus for insulin receptor substrate-1) produce significant insulin resistance in mice while any single-locus heterozygotes alone cannot [28, 30]. Hoh and Ott [28] have reviewed a few examples of genotypic interactions with 100% penetrance, suggesting the presence of zygotic LD at the population level. Many complex diseases, fitness-related traits, might involve epistasis within genes or between genes, such as diabetes, cystic fibrosis, cardiovascular and neurodegenerative diseases [3, 29, 31, 32, 33]. It is of medical significance to map epistasis to specific regions at the DNA (epistatic SNPs), transcriptional RNA, and translational protein levels.

The purpose of this study is to (i) investigate the patterns of zygotic LDs in human populations that are focused on in the International HapMap Project, and (ii) search for genome-wide epistatic SNPs that are associated with fitness. These focal populations have distinct ancient originations and represent a broad genetic variation in *H*. *sapiens*. Through investigating the patterns of zygotic LDs, we corroborate the utility of zygotic LD in characterizing human population history [15]. By comparing the maximum zygotic LD with the composite digenic LD, we search for common epistatic SNPs in four Africa populations, three Asia populations, and two Europe populations. Because the method is not based on a specific disease trait but on the fitness only, the detected epistatic SNPs are likely associated with common diseases that have been clinically verified or unverified yet in different geographical regions. The findings are of potential significance for suggesting therapeutic strategies of common life-limiting disorders or for genomic medicine.

## Materials and Methods

### Genotyping samples

Genotyping data of eleven human populations are obtained from website http://hapmap.ncbi.nlm.nih.gov (Phase 3 release #3, NCBI build 36, dbSNP b126), which is released by The International HapMap Consortium [34]. Population samples are detailed in the literature [34, 35, 36]. SNP genotypes are generated using the platforms of the Illumina Human1M (by the Wellcome Trust Sanger Institute) and the Affymetrix SNP 6.0 (by the Broad Institute). These populations consist of ASW(Africa ancestry in Southwest USA) with the sample size *n* = 87, CEU (Utah residents with Northern and Western European ancestry from the CEPH collection) with *n* = 165, CHB (Han Chinese in Beijing, China) with *n* = 137, CHD (Chinese in Metropolitan Denver, Colorado) with *n* = 109, GIH (Gujarati Indians in Houston, Texas) with *n* = 101, JPT (Japanese in Tokyo, Japan) with *n* = 113, LWK (Luhya in Webuye, Kenya) with *n* = 110, MEX (Mexican ancestry in Los Angeles, California) with *n* = 86, MKK (Maasai in Kinyawa, Kenya) with *n* = 184, TSI (Toscans in Italy) with *n* = 102, and YRI (Yoruba in Ibadan, Nigeria) with *n* = 203. The genetic relatedness among individuals in each population is recorded in document relationships_w_pops_041510.txt (http://www.mmnt.net/db/0/0/ftp.ncbi.nih.gov/hapmap/genotypes/2010-05_phaseIII).

From the International HapMap Consortium, all online population SNPs have passed the quality control (QC) [34, 35, 36]. Here, we filter those SNPs (based on rs ID) that are not shared among the eleven populations for each chromosome. The numbers of common SNPs on each chromosome are shown in Table 1. In addition, according to SNP allele frequencies separately estimated in each population, we further filter all the SNPs with the minor allele frequencies (MAF) being smaller than 5% in each population.

**Table 1:** Summary of the common SNPs among eleven populations, the mean total SNP pairs (±Sd) within 10Mb and their ranges among eleven populations, and the mean total SNP pairs (±Sd) each with at least one significant zygotic LD (p-value <10^−9^) and their ranges among eleven populations.

Chr	Common SNPs	Mean total SNP pairs	Range	Mean total zygotic LDs	Range
1	84211	1192146±202348.4	824576–1546225	588126.3±161102.1	343909–864922
2	86440	1239791±219419.5	809047–1583733	602808.3±171506.5	324909–883086
3	72115	1039618±180417	669379–1306751	506805.5±139753.3	274642–740673
4	64432	857091.5±149644.6	576443–1091943	418699.2±114136.8	234711–606861
5	66291	883108.7±188055.7	591090–1212940	423403.8±160738.3	136248–673758
6	68620	1188641±211311.2	842170–1602582	555256.9±151774.9	331637–826120
7	57087	774898.5±116540.1	555833–951972	375182.5±94693.79	227009–524667
8	56246	860847.2±156796.3	560370–1110507	406868.3±111584	222346–590176
9	47911	654781.3±109010	466281–816269	304463.8±78319.78	179607–431718
10	54681	774483.2±127979.8	508795–958939	365102±94288.46	206361–517474
11	52879	809600.5±154265.5	545571–1062220	394045.7±117039.7	207825–587339
12	50273	663968.2±104969.5	466512–827071	314055±80741.9	186732–455120
13	39000	516537±71580.25	399799–637859	242179.3±61410.3	152920–343475
14	33981	504617.4±79977.97	345940–622227	253895.6±60392.86	149834–329228
15	31404	404004.5±71633.31	279268–518707	193767±55883.93	108534–289225
16	32362	405996.6±60844.55	296450–504439	188003.3±46070.99	113379–270404
17	27717	342608.3±56911.77	241241–447118	175707.3±47083.35	102010–256577
18	30730	368843.6±53599.91	262473–470115	175166±45583.89	103239–256476
19	19401	198264.5±27020.22	145348–241725	96462.36±22699.96	60196–131683
20	26698	348270.7±49774.02	261096–424359	163964.3±40246.45	101216–233946
21	15008	179983±30755.05	126992–226505	83953.73±23479.56	48395–121520
22	14481	170236.2±26370.43	115980–200872	80403.64±19094.54	46461–108334

To simplify zygotic LD analysis, SNP genotypes are re-coded by one diallelic locus (say, alleles *A* and *a*) in each population in the following way. First, we set the genotypes of the individuals with nucleotide bases A/C, A/G, and A/T as heterozygote *Aa*, the genotypes for the individuals with nucleotide bases G/G, C/C, and T/T as homozygote *aa*, and the genotypes of the individuals with nucleotide bases A/A as homozygote *AA*. Then, we set the genotypes of the individuals with nucleotide bases C/G and C/T as heterozygote *Aa*, the genotypes of the individuals with nucleotide bases G/G and T/T as homozygote *aa*, and the genotypes of the individuals with nucleotide bases C/C as homozygotes *AA*. Finally, we set the genotypes of the individuals with nucleotide bases G/T as heterozygote *Aa*, the genotypes of the individuals with nucleotide bases G/G as homozygote *AA*, and the genotypes of the individuals with nucleotide bases T/T as homozygote *aa*. The information on individual nucleotide bases (A, T, G, C) at each locus is not recorded in analysis.

### Statistical tests

Because genome-wide gametic LDs have already been extensively examined in the HapMap populations in separate studies [34, 35, 36], we do not further recalculate them. We concentrate on zygotic LD and its comparison with the composite digenic LD. Consider two diallelic loci A and B, with alleles *A* and *a* at locus A and *B* and *b* at locus B. Let ${\hat{p}}_{ijkl}$ be the maximum likelihood estimate (MLE) of the frequency of genotype *ijkl* (*i*, *j* = *A*, *a*; *k*, *l* = *B*,*b*) in a population, and ${\hat{p}}_{ij}$ (or ${\hat{p}}_{kl}$) be the MLE of the frequency of genotype *ij* at locus A (or *kl* at locus B) in the population. MLEs of genotypic and allelic frequencies are obtained by the directly counting method from samples [25]. Let *D*_*ijkl*_ be the zygotic LD between genotypes *ij* at locus A and *kl* at locus B for genotype *ijkl*, which is estimated by

$$\begin{array}{ll} {\hat{D}}_{ijkl} & ={\hat{p}}_{ijkl}-{\hat{p}}_{ij}{\hat{p}}_{kl},\\ & =n_{ijkl}/n-n_{ij}n_{kl}/n^{2} \end{array}$$ (1)

where *n*_*ijkl*_, *n*_*ij*_, and *n*_*kl*_ are the numbers of genotypes *ijkl* (*i*, *j* = *A*, *a*; *k*, *l* = *B*,*b*), *ij* (*i*, *j* = *A*, *a*), and *kl* (*k*, *l* = *B*,*b*) in the sample of size *n*.

For two diallelic loci, there are nine zygotic LDs in total, but only four of them are random because of the following constraints: *D*_*AAkl*_+*D*_*Aakl*_+*D*_*aakl*_ = 0, (*k*,*l* = *B*,*b*) and *D*_*ijBB*_+*D*_*ijBb*_+*D*_*ijbb*_ = 0 (*i*,*j* = *A*, *a*). Here, we focus on four zygotic LDs, i.e. *D*_*AABB*_, *D*_*AABb*_, *D*_*AaBB*_, and *D*_*AaBb*_. The rest five zygotic LDs are constrained among the nine zygotic LDs. Note that there is one error in describing the total zygotic LDs (nine, not eight) and also an imprecise description of independence of the four zygotic LDs in [15, 21]. The four zygotic LDs are correlated owing to their sharing of common alleles in the same samples, which can be seen from the covariances between distinct zygotic LDs, e.g., Eq (2) in [15]

Hu and Yeh [21] have derived chi-square statistics to test the four zygotic LDs. Analogous to the commonly used *r* ^2^ for the normalized gametic LD (= $D_{AB}^{2}/{\hat{p}}_{A}{\hat{p}}_{a}{\hat{p}}_{B}{\hat{p}}_{b}$ = χ^2^_*AB*_/2*n* where χ^2^_*AB*_ is the chi-square statistic with one degree of freedom and ${\hat{D}}_{AB}$ is the gametic LD), we use the normalized zygotic LDs derived from chi-square statistics. From Hu and Yeh [21], a chi-square statistic with one degree of freedom for testing null hypothesis H_0_: *D*_*AjBl*_ = 0 (*j* = *A*, *a*; *l* = *B*, *b*) is set as:

$$\chi_{AjBl}^{2}=\frac{n{\hat{D}}_{AjBl}^{2}}{{\hat{p}}_{Aj}(1-{\hat{p}}_{Aj}){\hat{p}}_{Bl}(1-{\hat{p}}_{Bl})}.$$ (2)

The normalized zygotic LD is set as

$$r_{AjBl}^{2}=\chi_{AjBl}^{2}/n,\;\text{(}j=A,a\text{;}\;l=B,b\text{)}.$$ (3)

The r-square, *r* ^2^_*AjBl*_, ranges from 0 to 1.

The composite digenic LD, denoted by *Δ*_*AB*_, is 2*p*_*AABB*_+*p*_*AABb*_+*p*_*AaBB*_+*p*_*AaBb*_/2-2*p*_*A*_ *p*_*B*_ [25], which can be estimated in terms of zygotic LDs: ${\hat{\Delta }}_{AB}={\hat{D}}_{AABB}+{\hat{D}}_{AABb}+{\hat{D}}_{AaBB}+{\hat{D}}_{AaBb}/2$ [21]. Note that the preceding expression is alternative to Weir’s formula derived as the sum of gametic LD (*D*_*AB*_) and the disequilibrium (*D*_*A/B*_) between two non-alleles from different gametes within individuals, i.e. ${\hat{\Delta }}_{AB}={\hat{D}}_{AB}+{\hat{D}}_{A/B}$ [25]. Weir’s formula explicitly indicates that the composite digenic LD remains a low-order LD but is always not smaller than the gametic LD.

From Hu and Yeh [21], a chi-square statistic with one degree of freedom for testing null hypothesis H_0_: *Δ*_*AB*_ = 0 is set as

$$\chi_{\Delta }^{2}=\frac{n{\hat{\Delta }}_{AB}^{2}}{V({\hat{\Delta }}_{AB})}.$$ (4)

The variance of composite digenic LD is given in Hu and Yeh [21],

$$\begin{array}{l} V({\hat{\Delta }}_{AB})=\frac{1}{n}({\hat{\Delta }}_{AB}(1-{\hat{\Delta }}_{AB}-2({\hat{p}}_{A}+{\hat{p}}_{B}-4{\hat{p}}_{A}{\hat{p}}_{B}))+({\hat{p}}_{A}{\hat{p}}_{a}+{\hat{D}}_{A})({\hat{p}}_{B}{\hat{p}}_{b}+{\hat{D}}_{B})\\ \;+2{\hat{D}}_{AABB}-{\hat{D}}_{AaBb}/4-4{\hat{p}}_{A}({\hat{D}}_{AABB}+{\hat{D}}_{AaBB}/2)-4{\hat{p}}_{B}({\hat{D}}_{AABB}+{\hat{D}}_{AABb}/2)) \end{array}$$ (5)

in which ${\hat{D}}_{A}={\hat{p}}_{AA}-{\hat{p}}_{A}^{2}$ and ${\hat{D}}_{B}={\hat{p}}_{BB}-{\hat{p}}_{B}^{2}$ are the Hardy-Weinberg disequilibrium (HWD) at loci A and B, respectively.

As indicated in [21], Eq (5) is different from Weir and Cockerham’s expression in that it is derived in terms of zygotic LDs (rather than gametic LD), HWD, and allele frequency [37]. The feature is that its calculation does not need to estimate gametic LD from diploid genotyping data under HWE or the random mating assumption. To test *Δ*_*AB*_ = 0 using Eq (4), ${\hat{\Delta }}_{AB}$ is set as zero in $V({\hat{\Delta }}_{AB})$. Zygotic LD and HWD are set as zero in $V({\hat{\Delta }}_{AB})$ if they are not significantly different from zero. Zygotic LD is tested using Eq (2), and HWD is tested using chi-square statistic [25]. Similarly, the normalized composite digenic LD is set as *r* ^*2*^_*Δ*_ = *χ* ^2^_Δ_/*n*, which ranges from 0 to 1.

To test the difference between |Δ_*AB*_| and the maximum absolute zygotic LD, i.e. $\hat{d}=\left|{\hat{\Delta }}_{AB}\left|-\text{max}(\right|{\hat{D}}_{AABB}\left|,\;\right|{\hat{D}}_{AABb}\left|,\;\right|{\hat{D}}_{AaBB}\left|,\;\right|{\hat{D}}_{AaBb}\right|)$, from Hu and Yeh [21], a chi-square statistic with one degree of freedom for testing null hypothesis H_0_: *d* = 0 is set as

$$\chi_{AjBl(-)}^{2}=\frac{{\hat{d}}_{AjBl(-)}^{2}}{V({\hat{d}}_{AjBl(-)})},$$ (6a)

or

$$\chi_{AjBl(+)}^{2}=\frac{{\hat{d}}_{AjBl(+)}^{2}}{V({\hat{d}}_{AjBl(+)})},\;\text{(}j=A,a;\;l=B,b\text{)}$$ (6b)

where $V({\hat{d}}_{AjBl(-)})$ or $V({\hat{d}}_{AjBl(+)})$ is detailed in Eq (24) of Hu and Yeh [21]. Note that the maximum zygotic LD may be a different one of the four zygotic LDs in different SNP pairs, and its sign may be positive or negative. The composite digenic LD may be positive or negative as well. The subscript *AjBl(-)* in Eq (6a) refers to the case where both *Δ*_*AB*_ and the maximum zygotic LD are positive or both are negative. The subscript *AjBl(+)* in Eq (6b) refers to the case where *Δ*_*AB*_ and the maximum zygotic LD are different in sign.

When *d* for a SNP pair is significantly greater than zero, no epistatic selection is indicated between the two SNPs [15, 21]. The normalized r-square for *d>*0 is denoted as *r* ^2^_*d>0*_ that equals *χ* ^2^_*AjBl*(-)_/*n* or *χ* ^2^_*AjBl*(+)_/*n* with *d*>0. When *d* for a SNP pair is significantly smaller than zero, strong epistatic selection is indicated between the two SNPs [15, 21]. The normalized r-square for *d<*0 is set as *r* ^2^_*d<0*_ that equals *χ* ^2^_*AjBl*(-)_/*n* or *χ* ^2^_*AjBl*(+)_/*n* with *d*<0. These r-squares range from 0 to 1.

Note that a caution is needed in applying the above methods to testing zygotic and composite digenic LDs, and their differences. Because the variances of the parameters are derived using Fisher’s delta method [25] in constructing chi-square statistics or z-scores [21], a large sample size is required in order to obtain good approximation of variance. When MAF is too small or the sample size is too small, say *n*<30, unstable test results could be produced, which is similar to testing gametic LD using the chi-square statistic [25].

In our calculations, we confine our analysis to syntenic SNP pairs within 10Mb (~10 cM in human genomes) so as to make the data be manageable. Based on Weir and Cockerham’s [37] suggestion, the steps for statistical tests used by Hu and Yeh [21] for each population are summarized as follows. First, test individual zygotic LDs for pairwise SNPs using Eq (2). Note that the SNP with MAF<5% is excluded. Then, test the composite digenic LD. *Δ*_*AB*_ in $V({\hat{\Delta }}_{AB})$ is set as zero. The specific zygotic LD in $V({\hat{\Delta }}_{AB})$ is dropped if it is not significantly different from zero. HWD at each locus in $V({\hat{\Delta }}_{AB})$ is dropped if it is not significantly different from zero. The composite digenic LD for a SNP pair is tested only when at least one of the four zygotic LDs is significant, which otherwise the test is not conducted further for this SNP pair. Finally, test the difference between the composite digenic and maximum zygotic LDs. The *d* value in $V(\hat{d})$ using Eq (6) is set as zero, and non-significant zygotic LDs in $V(\hat{d})$ are dropped. Similarly, the test is conducted only when the SNP pair has at least one significant zygotic LD.

The above analysis is separately conducted on each chromosome in each population. To derive more conservative conclusions, a very stringent significant level for each test is set as p-value<10^−9^, smaller than the Bonferroni adjusted p-value (0.01/ the maximum number of SNP pairs). All these calculations and tests are conducted with a program in C used by Hu and Yeh [21].

To investigate the pattern of the zygotic and composite digenic LDs along chromosomes, we test Pearson’s correlation between the significant *r* ^2^_*AjBl*_ (*j* = *A*, *a*; *l* = *B*, *b*) or *r* ^2^_*Δ*_ and the physical distance over all autosomes in each population. The correlations between the significant *r* ^2^_*d>0*_ or *r* ^2^_*d<0*_ and the physical distance are tested as well. All these tests are conducted in R.

Hierarchical cluster analysis among the eleven populations is conducted using the joint information, including the total and the average r-squares of significant zygotic and composite digenic LDs, the average physical distances for significant r-squares, and Pearson’s correlations between the significant r-square and the physical distance. Population distance matrix is constructed according to the algorithm of Canberra distance between any two vectors [38]. R-function, pvclust (), is applied for plotting [39].

## Results

### Total zygotic LDs

The number of common SNPs among the eleven populations generally decreases from Chromosome (Chr) 1 to 22 (Table 1). Chr 2 has the most common SNPs (86440) while Chr 22 has the fewest common SNPs (14481). The total SNP pairs within 10Mb (with MAF≥5% at each SNP locus) display the pattern similar to that of the number of common SNPs, with the longer chromosome harbouring more SNP pairs (Table 1). From the statistical tests of zygotic LDs (H_0_: *D*_*AjBl*_ = 0; *j* = *A*, *a*; *l* = *B*, *b*), the total SNP pairs with at least one significant zygotic LD generally decrease from Chr 1 to 22. Chr 2 has the most significant zygotic LDs (324909–883086 in different populations). On average, about 47.4% (±6.8%) SNP pairs each with MAF ≥5% within 10Mb per chromosome per population (a range of 23.1~59.3%) have at least one significant zygotic LD, indicating that extensive zygotic LDs exist in human populations.

**Fig 1:**
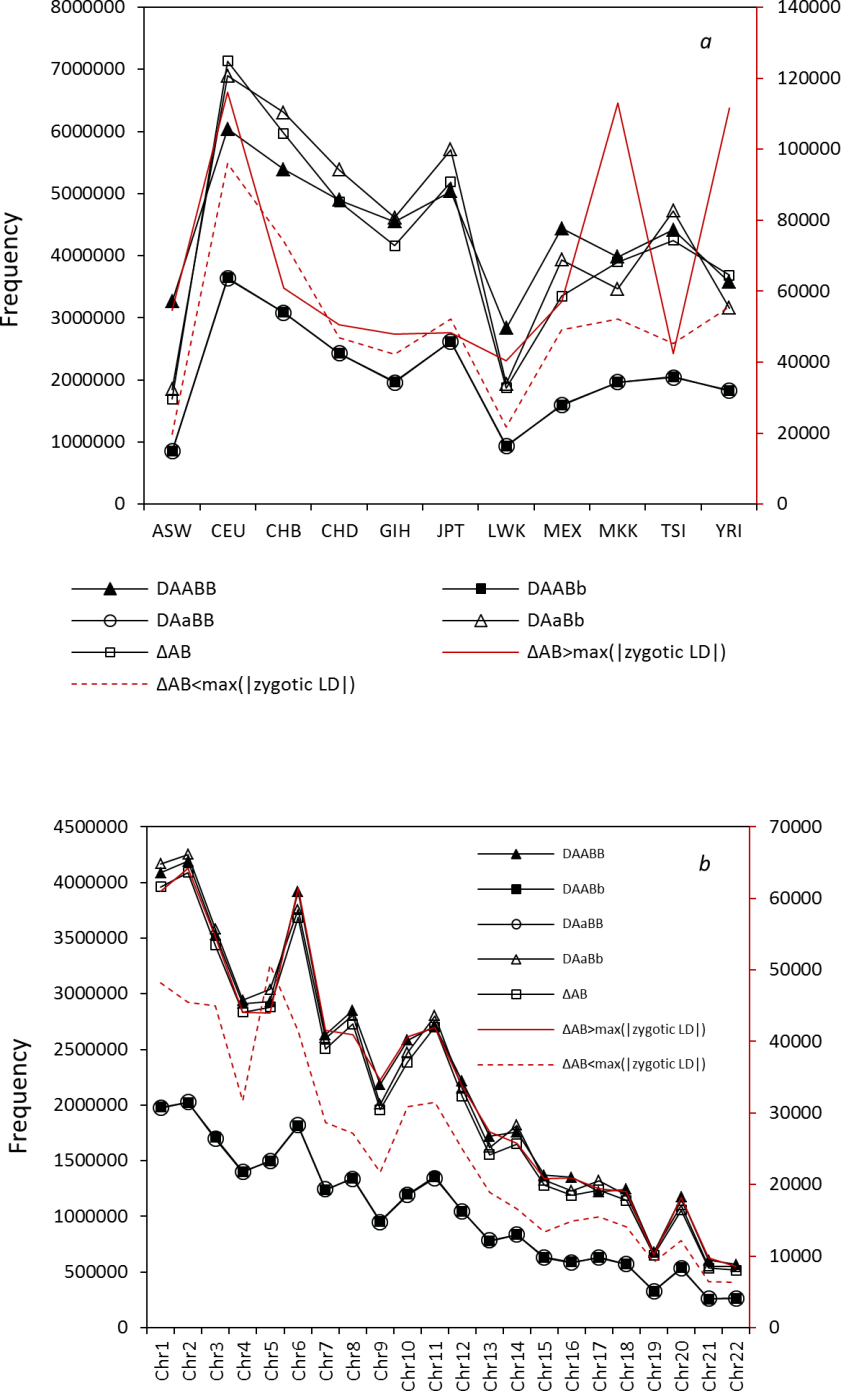
Total SNP pairs of the significant zygotic LDs, the significant composite digenic LDs, and the significant differences between the composite digenic and maximum zygotic LDs. (a) for each population over all autosomes; (b) for each autosome over all populations. The primary y-axis is used for all black lines while the secondary y-axis is used for the red solid and dashed lines.

The total significant zygotic LDs over all autosomes are unequal among populations or among four zygotic LDs in each population (Fig 1a). Population CEU has the most significant zygotic LDs for each of the four two-locus genotypes (*D*_*AjBl*_≠0, *j* = *A*, *a*; *l* = *B*,*b*), followed by populations CHB, JPT, CHD, TSI, GIH, MEX, and the four Africa populations (MKK, YRI, ASW, and LWK). With a reference to the specific zygotic LD, populations CEU, CHB, CHD, JPT, GIH, and TSI have more significant zygotic LDs for the double heterozygotes (*D*_*AaBb*_) than for other three zygotic LDs (*D*_*AABB*_, *D*_*AABb*_, and *D*_*AaBB*_); while populations ASW, LWK, MEX, MKK, and YRI have more significant zygotic LDs for the double homozygotes (*D*_*AABB*_) than for other three zygotic LDs (*D*_*AABb*_, *D*_*AaBB*_, and *D*_*AaBb*_). There are fewer significant zygotic LDs for the homozygote-heterozygote genotypes (*D*_*AABb*_ or *D*_*AaBB*_) than for other two zygotic LDs (*D*_*AABB*_ and *D*_*AaBb*_) in each population.

Population CEU has the most significant composite digenic LDs (*Δ*_*AB*_≠0) over all autosomes, followed by populations CHB, JPT, CHD, TSI, GIH, MKK, YRI, and MEX. Two Africa populations, LWK and ASW, have a relatively fewer significant composite digenic LDs (Fig 1a). Like the patterns of *D*_*AABB*_ and *D*_*AaBb*_, population MEX has the fewest SNP pairs with significant composite digenic LDs among the seven non-Africa populations.

A significant correlation exists between the total significant zygotic LDs and the number of common SNPs, or between the total significant composite digenic LDs and the number of common SNPs across all autosomes (Pearson’s correlations = 0.99, p-value<10^−16^; Fig 1b). However, Chrs 6, 11, and 20 have relatively more significant zygotic LDs or more significant composite digenic LDs; while Chrs 3, 4, and 19 have a fewer significant zygotic LDs or a fewer significant composite digenic LDs. A common feature is that each autosome has more significant *D*_*AABB*_ ‘s or *D*_*AaBb*_’s or *Δ*_*AB*_’s than significant *D*_*AABb*_’s or *D*_*AaBB*_’s.

Concerning the difference between the composite digenic and maximum zygotic LDs $\left(\hat{d}\right)$, about 1.80% (±0.9%) of significant *Δ*_*AB*_’s, ranging from 0.93% to 3.23%, are significantly greater than the maximum zygotic LDs (*d*>0), indicating strong signals of linear additive effects on fitness. About 1.21% (±0.2%) of significant *Δ*_*AB*_’s, ranging from 0.96% to 1.51%, are significantly smaller than the maximum zygotic LDs (*d*<0), indicating strong signals of epistatic selection for fitness. A majority of the composite digenic LDs are comparable to the maximum zygotic LDs in magnitude, indicating weak signals of epistatic selection [15], [21]. Populations CEU, MKK, and YRI have more SNP pairs displaying significant *d*(>0) than do the rest populations (Fig 1a), indicating that the three populations have relative more SNPs pairs strongly exhibiting non epistatic effects on population fitness than do the rest populations (LWK, ASW, CHD, CHB, GIH, JPT, MEX and TSI). Population CEU has the most SNP pairs with the *d* values being significantly smaller than zero (*d*<0), while two Africa populations (ASW and LWK) have the fewest SNP pairs with the *d* values being significantly smaller than zero (*d*<0; Fig 1a).

Pattern of the total SNP pairs with *d* >0 over all populations is similar to that of the total composite digenic LD (*Δ*_*AB*_) across all autosomes (Fig 1b). Each chromosome has more SNP pairs with *d* >0 than those with *d* <0 except Chr 5. The main reason for more SNP pairs with *d*<0 on Chr 5 is because population MEX has more SNP pairs with *d*<0 on Chr 5 than do other populations (details not shown here).

### R-squares

**Fig 2:**
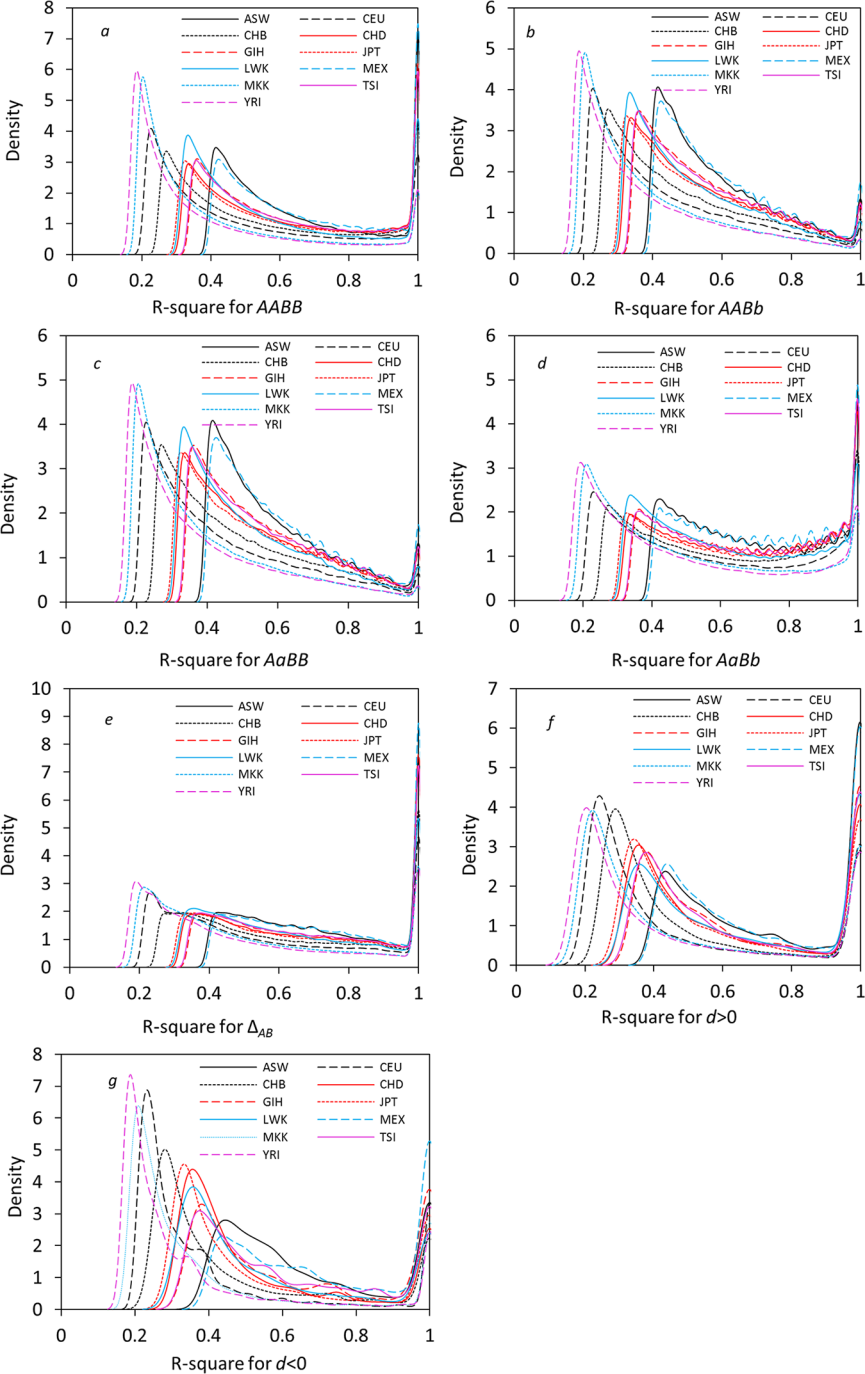
Density distributions of the significant r-squares in eleven populations. (a) *D*_*AABB*_; (b) *D*_*AABb*_; (c) *D*_*AaBB*_; (d) *D*_*AaBb*_; (e) the composite digenic LD; (f) the difference *d* >0; and (g) the difference *d*<0.

The distribution patterns of significant zygotic LDs over all autosomes are highly heterogeneous among populations (Mann-Whitney test for all pairwise populations, p-value<10^−16^). One common feature is a generally consistent order of distributions among eleven populations for the normalized zygotic LDs or the normalized composite digenic LDs (Fig 2a, 2b, 2c, 2d, 2e, 2f and 2g for the density distribution and Table 2 for means and standard deviations). The density distributions are bimodal, with one peak close to lower r-squares and another peak at high r-squares (= 1.0). All average r-squares (*r* ^2^_*AjBl*_ (*j* = *A*, *a*; *l* = *B*, *b*); *r* ^2^_*Δ*_, *r* ^2^_*d<0*_, and *r* ^2^_*d>0*_) increase generally in the order of YRI, MKK, CEU, CHB, LWK, JPT, CHD,TSI, GIH, ASW, and MEX. Although CEU has the most significant zygotic LDs (over all autosomes), it has relatively lower zygotic LDs. Although LWK and ASW have a relatively fewer significant zygotic LDs, their r-squares are not small (Table 2). Population MEX does not have the most significant zygotic LDs, but has high zygotic LDs, indicating that the number of significant zygotic LDs does not reflect their strengths.

**Table 2:** Means and standard deviations over all autosomes for the r-squares of the significant zygotic LDs, the r-squares of the significant composite digenic LD, and the r-squares of the significant differences between the composite digenic and maximum zygotic LDs in each population (p-value <10^−9^).

Population	*r* ^2^_*AABB*_	*r* ^2^_*AABb*_	*r* ^2^_*AaBB*_	*r* ^2^_*AaBb*_	*r* ^2^_*Δ*_	*r* ^2^_*d>0*_	*r* ^2^_*d<0*_
ASW	0.6623±0.2131	0.5937±0.1649	0.5936±0.1648	0.6971±0.1996	0.7112±0.2043	0.7383±0.2350	0.6688±0.2182
CEU	0.5002±0.2623	0.4448±0.2058	0.4444±0.2058	0.5740±0.2673	0.5500±0.2642	0.4927±0.2982	0.4120±0.2553
CHB	0.5644±0.2560	0.4941±0.1993	0.4945±0.1995	0.6193±0.2492	0.6071±0.2514	0.5302±0.2802	0.4842±0.2561
CHD	0.6305±0.2382	0.5517±0.1858	0.5522±0.1861	0.6712±0.2252	0.6663±0.2313	0.6360±0.2660	0.5479±0.2391
GIH	0.6396±0.2300	0.5630±0.1762	0.5632±0.1764	0.6795±0.2177	0.6826±0.2239	0.6637±0.2584	0.6425±0.2533
JPT	0.6213±0.2434	0.5435±0.1898	0.5439±0.1895	0.6669±0.2320	0.6572±0.2369	0.6134±0.2686	0.5551±0.2606
LWK	0.5779±0.2307	0.5269±0.1792	0.5265±0.1788	0.6352±0.2256	0.6421±0.2291	0.6614±0.2681	0.5651±0.2453
MEX	0.6818±0.2093	0.6074±0.1655	0.6083±0.1658	0.7097±0.1931	0.7211±0.2018	0.7316±0.2337	0.7244±0.2244
MKK	0.4169±0.2419	0.3953±0.1962	0.3950±0.1961	0.5072±0.2609	0.5047±0.2639	0.4912±0.3068	0.3916±0.2582
TSI	0.6389±0.2305	0.5649±0.1796	0.5646±0.1798	0.6809±0.2197	0.6759±0.2233	0.6614±0.2606	0.6267±0.2423
YRI	0.4006±0.2489	0.3826±0.2041	0.3824±0.2040	0.4956±0.2715	0.4872±0.2730	0.4725±0.3114	0.3594±0.2513

The average r-squares for significant zygotic LDs of the double heterozygotes (*r* ^2^_*AaBb*_) or the double homozygotes (*r* ^2^_*AABB*_) over all autosomes are greater than the average r-squares for significant zygotic LDs of the homozygote-heterozygote genotypes (*r* ^2^_*AABb*_ and *r* ^2^_*AaBB*_). Density distributions of *r* ^2^_*AABb*_ and *r* ^2^_*AaBB*_ are essentially identical (Fig 2b and 2c). The average r-squares for significant composite digenic LDs (*r* ^2^_*Δ*_) are large in each population, similar to the average *r* ^2^_*AaBb*_’s, but *r* ^2^_*Δ*_’s have different density distributions from those of *r* ^2^_*AaBb*_’s, with large densities close to 1 (Table 2; Fig 2e).

The average r-squares are generally greater for significant *d*>0 than for significant *d*<0 in addition to their distinct density distributions in each population (Table 2; Fig 2f and 2g).

Concerning with the physical distances between SNP pairs with at least one significant zygotic LD (Table 2), variations exist among populations or among the four zygotic LDs (Table 3). Most standard deviations are greater than means, with the coefficient of variation (CV) being greater than 1, which indicates quite variable distributions in physical distance. The common pattern is that the SNP pairs with significant *r* ^2^_*AABB*_ generally occur within longer physical distances (0.75~2.67Mb on average) than do the SNPs pars with significant *r* ^2^_*AABb*_ and *r* ^2^_*AaBB*_ (< 0.55 Mb on average), which in turn occurs within longer distances than the SNP pairs with significant *r* ^2^_*AaBb*_ and *r* ^2^_*Δ*_ (< 0.2 Mb on average). R-squares for significant *d* >0 occur within relatively longer distances (0.6~3.9 Mb on average) than those for significant *d*<0 (< 2 Mb on average).

**Table 3:** Means and coefficients of variations (standard deviation/mean) over all autosomes for the physical distance (Mb) of the significant zygotic LDs, the significant composite digenic LD, and the significant differences between the composite digenic and maximum zygotic LDs in each population (p-value <10^−9^).

Population	*AABB*	*AABb*	*AaBB*	*AaBb*	*Δ_AB_*	*d>0*	*d<0*
ASW	2.6687(1.17)	0.5380(3.14)	0.5268(3.18)	0.0656(4.61)	0.1653(4.89)	3.9368(0.79)	1.3636(1.89)
CEU	0.7519(2.61)	0.1287(4.59)	0.1233(4.51)	0.0978(3.80)	0.1093(3.57)	0.6645(2.78)	0.2656(4.19)
CHB	0.9439(2.33)	0.1294(4.79)	0.1259(4.83)	0.0922(3.93)	0.0973(3.79)	0.8288(2.49)	0.4892(3.41)
CHD	1.1975(2.04)	0.1429(5.00)	0.1434(5.01)	0.0774(3.49)	0.0859(3.59)	2.1220(1.42)	0.7599(2.65)
GIH	1.3090(1.93)	0.1562(4.94)	0.1504(5.01)	0.0746(3.58)	0.0855(3.96)	2.1149(1.40)	0.9377(2.38)
JPT	1.0701(2.17)	0.1403(4.82)	0.1391(4.75)	0.0843(3.38)	0.0940(3.49)	1.6048(1.69)	0.6483(2.88)
LWK	2.2058(1.38)	0.2965(4.14)	0.3141(4.08)	0.0537(3.65)	0.0713(4.84)	3.0638(1.04)	1.7147(1.67)
MEX	1.6860(1.64)	0.2511(4.34)	0.2521(4.35)	0.0702(3.44)	0.1278(4.50)	3.2485(0.97)	0.3929(3.70)
MKK	1.2918(1.94)	0.1987(4.56)	0.1959(4.55)	0.0807(4.03)	0.1041(4.29)	1.2240(1.99)	0.5006(3.31)
TSI	1.2011(2.04)	0.1379(5.06)	0.1365(5.05)	0.0758(3.91)	0.0873(4.03)	2.0765(1.44)	0.9105(2.44)
YRI	1.1632(2.08)	0.1431(5.06)	0.1418(5.09)	0.0702(3.67)	0.0812(3.79)	0.6101(2.93)	0.4432(3.53)

Populations vary in average physical distance among the four zygotic LDs. For the double homozygotes, the four Africa populations (YRI, MKK, LWK, and ASW) generally have significant *r* ^2^_*AABB*_ within relatively longer distances (1.16~2.66 Mb), but population CEU has significant *r* ^2^_*AABB*_ within relatively shorter distances (0.75 Mb). The rest populations generally have significant *r* ^2^_*AABB*_ within the intermediate distances (Table 3). For the double heterozygotes, the four Africa populations have significant *r* ^2^_*AaBb*_ generally within shorter distances (0.053~0.08 Mb), but population CEU has significant *r* ^2^_*AaBb*_ within relatively longer distances (<0.1 Mb). For the homozygote-heterozygote genotypes, the four Africa populations generally have significant *r* ^2^_*AABb*_ or *r* ^2^_*AaBB*_ within relatively longer distances (0.14~0.53 Mb), while population CEU has significant *r* ^2^_*AABb*_ or *r* ^2^_*AaBB*_ within ~0.12 Mb on average. The rest populations have significant *r* ^2^_*AABb*_ or *r* ^2^_*AaBB*_ within the intermediate distances (~0.14 Mb on average).

The physical distance for significant *r* ^*2*^_*Δ*_ is the longest on average in population ASW (~0.16 Mb), followed by MEX (~0.12 Mb), CEU and MKK (~0.1Mb), and the rest populations (<0.1Mb). This order is distinct from the population order for any significant zygotic LDs. The average physical distances are longer for significant *r* ^2^_*Δ*_ than for significant *r* ^2^_*AaBb*_, but are shorter than for the r-squares of any other significant zygotic LDs (*r* ^2^_*AABB*_, *r* ^2^_*AaBB*_, and *r* ^2^_*AABb*_) in each population (Table 3).

The physical distances are quite variable for significant *r* ^2^_*d>0*_ and *r* ^2^_*d<0*_, with CV>1.0 in all populations except ASW (CV = 0.79) and MEX (CV = 0.97) for *r* ^2^_*d>0*_ (Table 3). The average distances for significant *r* ^2^_*d>0*_ are almost twice as long as those for significant *r* ^2^_*d<0*_ in each population. Populations ASW, LWK, and MEX have significant *r* ^2^_*d>0*_ within longer distances (>3 Mb), while populations CEU and YRI have significant *r* ^2^_*d>0*_ within relatively shorter distances (~0.6 Mb). The four Africa populations can be separated by their average physical distances of significant *r* ^2^_*d<0*_, with long distances in ASW and LWK (<2 Mb) but short distances in YRI and MKK (~0.5 Mb). Populations CEU and MEX have significant *r* ^2^_*d<0*_ within shorter distances (<0.5 Mb on average), but have large variations (CV>3.0). The physical distances for significant *r* ^2^_*d<0*_’s are intermediate (0.5~1.0 Mb on average) in the rest populations (Table 3).

### Patterns of r-squares along chromosomes

Pearson’s correlation tests indicate that r-squares of the four zygotic LDs are significantly negatively correlated with the physical distance (Table 4). The r-squares of the double homozygote LDs, *r* ^2^_*AABB*_’s, are more strongly correlated with the physical distance (Mb) than do the r-squares of other three zygotic LDs in each population except ASW that exhibits a relatively weaker correlation. Although there are significantly different distributions among populations in *r* ^2^_*AaBb*_, comparable correlations among them exist between *r* ^2^_*AaBb*_ and the physical distance (Pearson’s correlation ~ -0.1).

**Table 4:** Pearson’s correlations between the significant r-square and physical distance (Mb) across all autosomes in each population.

Population	*AABB*	*AABb*	*AaBB*	*AaBb*	*Δ_AB_*	*d>0*	*d<0*
ASW	-0.0913	-0.1394	-0.1397	-0.1078	-0.0255	0.0619	0.1968
CEU	-0.1931	-0.0841	-0.0860	-0.1017	-0.0630	0.0477	0.0340
CHB	-0.1945	-0.0981	-0.0972	-0.1004	-0.0526	0.0695	0.0527
CHD	-0.1802	-0.0986	-0.0987	-0.0954	-0.0494	0.1337	0.1389
GIH	-0.1685	-0.0976	-0.0966	-0.1041	-0.0442	0.0924	0.0567
JPT	-0.1845	-0.1030	-0.1003	-0.1074	-0.0546	0.0955	0.0766
LWK	-0.1368	-0.1386	-0.1383	-0.0948	-0.0470	0.1208	0.1826
MEX	-0.1229	-0.1048	-0.1019	-0.1178	-0.0235	0.1078	0.0536
MKK	-0.2045	-0.1326	-0.1325	-0.1134	-0.0673	0.0321	0.0669
TSI	-0.1625	-0.0813	-0.0866	-0.1011	-0.0284	0.1290	0.0901
YRI	-0.1937	-0.1097	-0.1092	-0.1039	-0.0545	-0.0139*	0.0830

*: P-value = 3.2×10^−6^

All statistical tests have p-value<10^−9^ except one test in population YRI (*d*>0).

**Fig 3:**
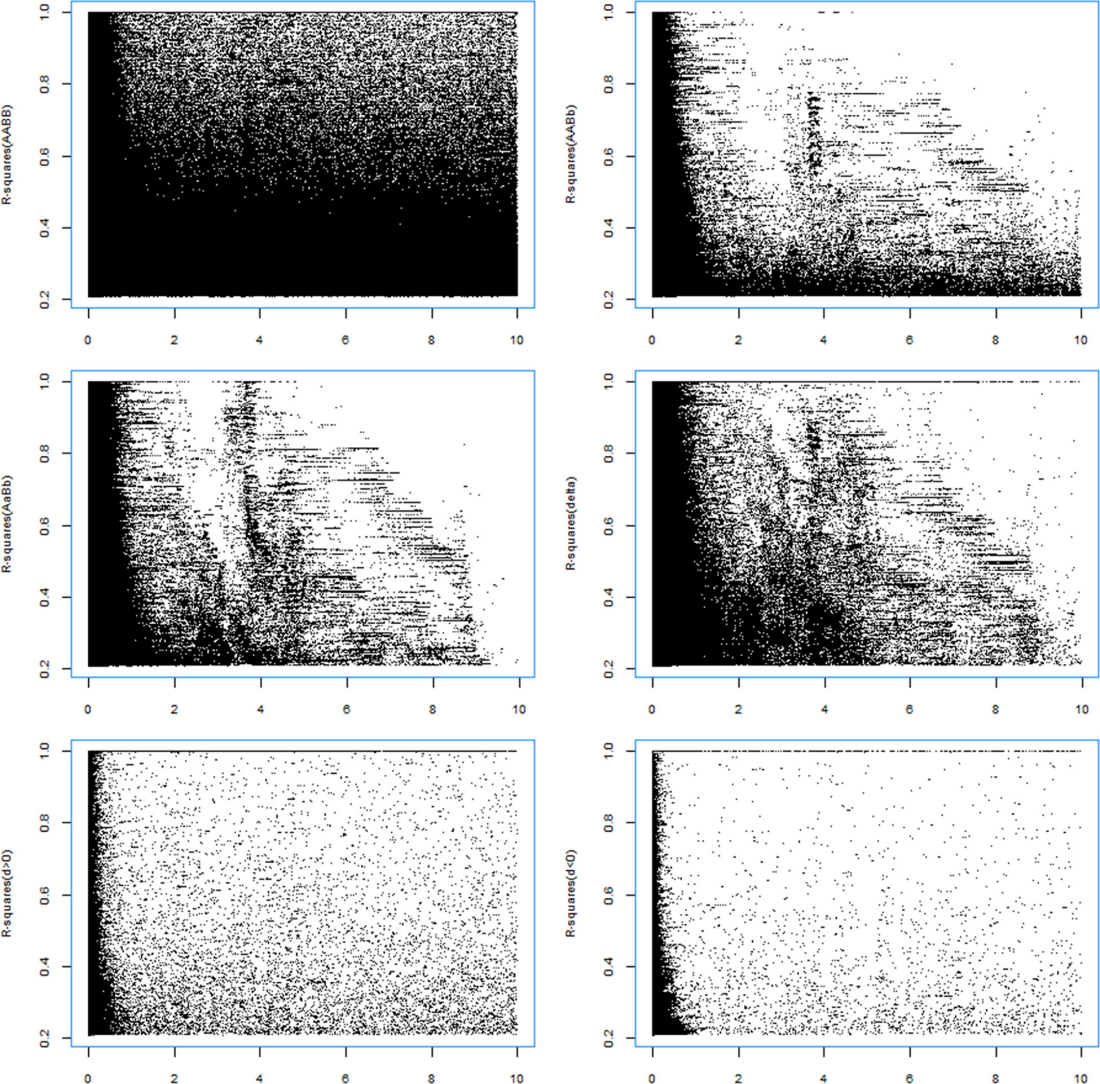
Pattern for the significant r-squares changing with the physical distances of SNP pairs (Mb) in population CEU. (a) *D*_*AABB*_ (6036652 pairs); (b) *D*_*AABb*_ (3647187 pairs); (c) *D*_*AaBb*_ (6893900 pairs); (d) the composite digenic LD (7130869 pairs); (e) the difference *d* >0 (116000 pairs); and (f) the difference *d*<0 (95921 pairs). The x-axis represents the physical distance between SNP pairs in Mb.

R-squares for the composite digenic LDs (*r* ^2^_*Δ*_) are also significantly negatively correlated with the physical distance (all p-values<10^−9^), but their strengths are not as strong as the corrections between *r* ^2^_*AjBl*_ (*j* = *A*, *a*; *l* = *B*, *b*) and the physical distance in each population (Table 4). For instance, although the significant *r* ^2^_*Δ*_ has the most SNP pairs (7130869) among the significant *r* ^2^_*AjBl*_, *r* ^2^_*d<0*_, and *r* ^2^_*d>0*_ in CEU, most *r* ^2^_*Δ*_’s are distributed within short distances (Fig 3), producing a relatively weaker correlation between *r* ^2^_*Δ*_ and the physical distance (Pearson correlation = -0.0630, p-value<2.2×10^−16^).

Unlike the patterns of *r* ^2^_*AjBl*_ (*j* = *A*, *a*; *l* = *B*, *b*) and *r* ^2^_Δ_, both *r* ^2^_*d<0*_ and *r* ^2^_*d>0*_ are significantly positively correlated with the physical distance except population YRI (Table 4), indicating that SNPs on distant positions trend to have both additive and epistatic effects on fitness. Populations ASW and LWK exhibit relatively stronger correlations between *r* ^2^_*d<0*_ and the physical distance than do populations MKK and YRI.

**Fig 4:**
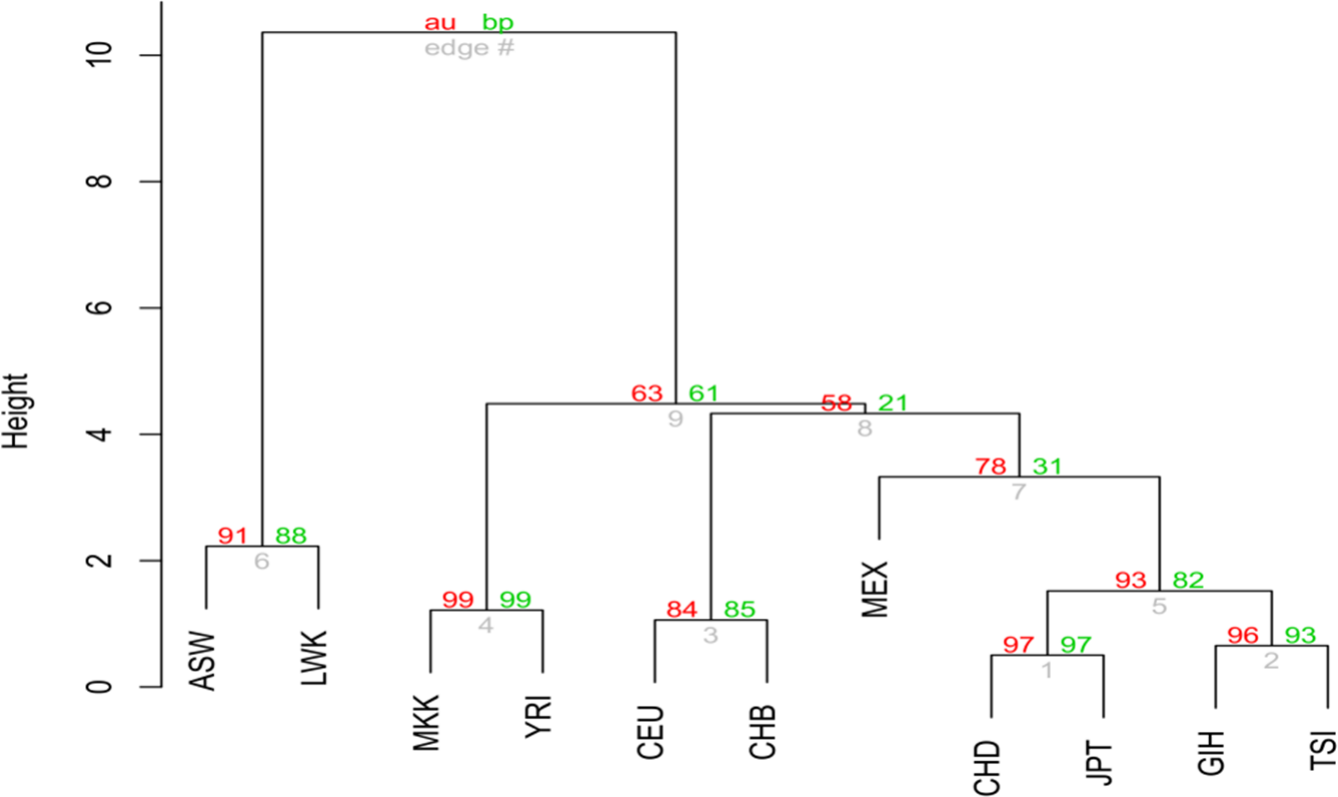
Cluster analysis of eleven human populations. The plot is based on the total significant zygotic and composite digenic LDs (Table 1), the average r-squares (Table 2), the average physical distances (Table 3), and Pearson’s correlations between the significant r-square and the physical distance (Table 4). Information on *d* (>0 or <0) is excluded in all tables. Distance matrix is constructed using Canberra distance between any two vectors (20-dimensional). The numbers in red are the p-values (out of 100) that are approximately unbiased (AU), and the numbers in green are the p-values (out of 100) derived from bootstrapping (BP).

Taken together all above information for cluster analysis (Fig 4), populations ASW and LWK (a probability of 91% in a group) can be separated from populations MKK and YRI (a probability of 99% in a group), with a probability of 63%. Four populations (CHD, JPT, GIH, and TSI) join together, with a probability of more than 90%. Population MEX can be considered as a separate group although it has a probability of 78% to join the group of CHD, JPT, GIH, and TSI. Populations CEU and CHB have a probability of 84% in a group, and join the group of all other non-Africa populations with a probability of more than 50%. Populations MKK and YRI have a probability of more than 60% to join the group of non-Africa populations, representing a “transitional” status from Africa populations ASW and LWK to non-Africa populations.

### Common epistatic SNPs

Initial screening indicates that no common SNP pairs of significant *r* ^2^_*d<0*_ are present among eleven populations. Most epistatic SNPs are distinct among populations or population specific. To capture the common SNPs among regions, we concentrate on four Africa populations (ASW, LWK, MKK, and YRI), three Asia populations (CHB, CHD, and JPT), and two Europe populations (CEU and TSI).

In the four Africa populations, a few chromosomes have common SNP pairs of significant *r* ^2^_*d<0*_, with two pairs on Chr 2, seventeen on Chr7, and two pairs on Chr 19 that show a strong signal of epistatic selection for population fitness (S1 Table). These significant *r* ^2^_*d<0*_’s range from 0.1642±0.0530 to 0.5356±0.4119, and the *d* values range from -0.0666±0.0203 to -0.0472±0.0156. One immediate feature is that these syntenic SNP pairs occur within 0.5 Mb. Gene annotations indicate 2 SNP pairs each with unknown function information, 9 SNP pairs where one SNP is annotated but the other is not annotated yet, 5 SNP pairs each within the same annotated genes, and 5 SNP pairs in three distinct gene pairs on Chr 7. The annotated genes involving the epistatic SNP pairs are VWC2L, von Willebrand factor C domain containing protein 2-like; PTPRN2, protein tyrosine phosphatase, receptor type, N polypeptide 2; ESYT2, extended synaptotagmin-like protein 2; CPAMD8, C3 and PZP-like, alpha-2-macroglobulin domain containing 8; and WDR60, WD repeat domain 60.

The three genes for the 5 SNP pairs on Chr 7 are PTPRN2, EYST2, and WDR60. They are located in the order PTPRN2-ESYT2-WDR60 on Chr 7, where five genes/microRNA transcripts are present in the interval between PTPRN2 and ESYT2 but none is present in the interval between ESYT2 and WDR60. SNP rs2335555 in the PTPRN2 gene, whose mutants induced by cigarette smoking can cause lung cancer, interacts with SNPs rs6459904 (*d* = -0.0493±0.0209, *r* ^2^_*d<0*_ = 0.2487±0.1848) and rs2952820 (*d* = -0.0523±0.0198, *r* ^2^_*d<0*_ = 0.1930±0.0970) in ESYT2 gene, whose mutants induced by sunlight/ultraviolet radiation cause malignant melanoma. Rs2335555 also interacts with rs1188974 (*d* = -0.0509±0.0209, *r* ^2^_*d<0*_ = 0.1880 ± 0.0913) in WDR60 gene, whose mutants cause the short-rib polydactyly and Jeune syndromes [40]. SNPs rs1188974 in WDR60 interacts with rs6459904 (*d* = -0.0642±0.0222, *r* ^2^_*d<0*_ = 0.1642±0.0530) and rs2788500 (*d* = -0.0510±0.0211, *r* ^2^_*d<0*_ = 0.2404±0.1482) in ESYT2, respectively.

**Fig 5:**
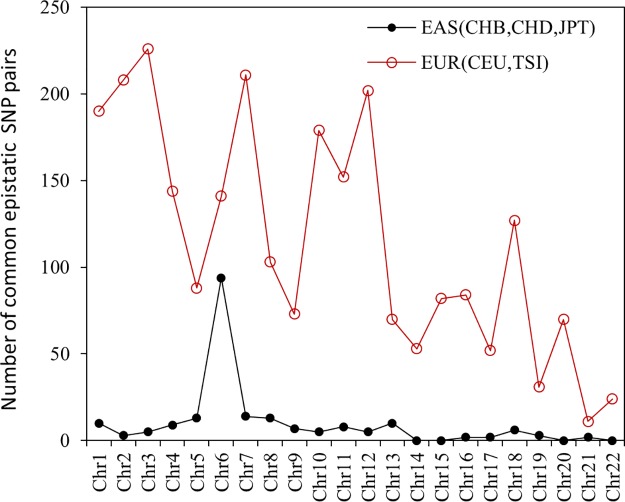
Common epistatic SNP pairs in East Asia (CHB, CHD, JPT) and in Europe (CEU and TSI) regions across all autosomes. Results are summarized from S2 and S3 Tables.

In the three Asia populations, there are 211 common SNP pairs of significant *r* ^2^_*d<0*_’s that are distributed on all autosomes except Chrs 14, 15, 20, and 22 (S2 Table). These *r* ^2^_*d<0*_’s range from 0.1724±0.1441 to 0.8636±0.2362, and the *d* values range from -0.1032±0.0020 to -0.0217±0.0130. Chr 6 has the most common SNP pairs (94; Fig 5). All physical distances are within 1 Mb (S2 Table), with the longest distance 896960 bp between rs1557580 (unknown annotation yet) and rs183395 (*d* = -0.0388±0.0066; *r* ^2^_*d<0*_ = 0.4216±0.085) in gene CADM1 (cell adhesion molecule 1) on Chr 12, and the shortest distance 26 bp between rs322790 and rs322780 on Chr7 (*d* = -0.0538±0.0062; *r* ^2^_*d<0*_ = 0.5199±0.1707). There are 75 SNP pairs where one SNP is annotated but the other is not in each pair, 72 SNP pairs where both SNPs are not annotated in each pair, 39 SNP pairs where each pair comes from the same annotated gene, and 25 SNP pairs where each pair comes from distinctly annotated genes (S2 Table).

Six gene-gene pairs in the 25 SNP pairs are involved in common epistatic selection for fitness. These include TTLL10 (tubulin tyrosine ligase-like family, member 10)- TNFRSF18 (tumor necrosis factor receptor superfamily, member 18); FGFR1OP(FGFR1 oncogene partner)- CCR6(chemokine (C-C motif) receptor 6); NUP153(nucleoporin 153kDa)- KIF13A (kinesin family member 13A); UBN2 (ubinuclein 2)- KLRG2 (killer cell lectin-like receptor subfamily G, member 2); LOC441242 (uncharacterized LOC441242)- VKORC1L1(vitamin K epoxide reductase complex, subunit 1-like 1); DOT1L(DOT1-like histone H3K79 methyltransferase)- JSRP1(junctional sarcoplasmic reticulum protein 1); and RWDD2B (RWD domain containing 2B)- USP16 (ubiquitin specific peptidase 16). These individual genes are directly or indirectly associated with human diseases and cancers, and can affect population fitness. However, clinic evidence for the common epistatic effects awaits further data collections.

In the two Europe populations (CEU and TSI), there are 2521 common SNP pairs of significant *r* ^2^_*d<0*_’s in total on all autosomes (S3 Table; Fig 5). Chr 3 has the most common SNP pairs (226) while Chr 21 has the fewest common SNP pairs (11). The physical distances of these epistatic SNPs are within 0.5 Mb, ranging from 16bp between rs500608 and rs3819114 on Chr 11 (*d* = -0.0625±0.0354; *r* ^2^_*d<0*_ = 0.4274±0.1897) to 338385bp between rs2522505 and rs17035289 on Chr2 (*d* = -0.0475±0.0283; *r* ^2^_*d<0*_ = 0.3433±0.0040). Compared with the results in the three Asia populations or in the four Africa populations, the *r* ^2^_*d<0*_ values of the common SNP pairs between populations CEU and TSI are higher, on average, with the mean ranging from 0.2768±0.0973 to 1.0±0.0. The *d* values range from -0.1086±0.0074 to -0.0131±0.0045.

**Fig 6:**
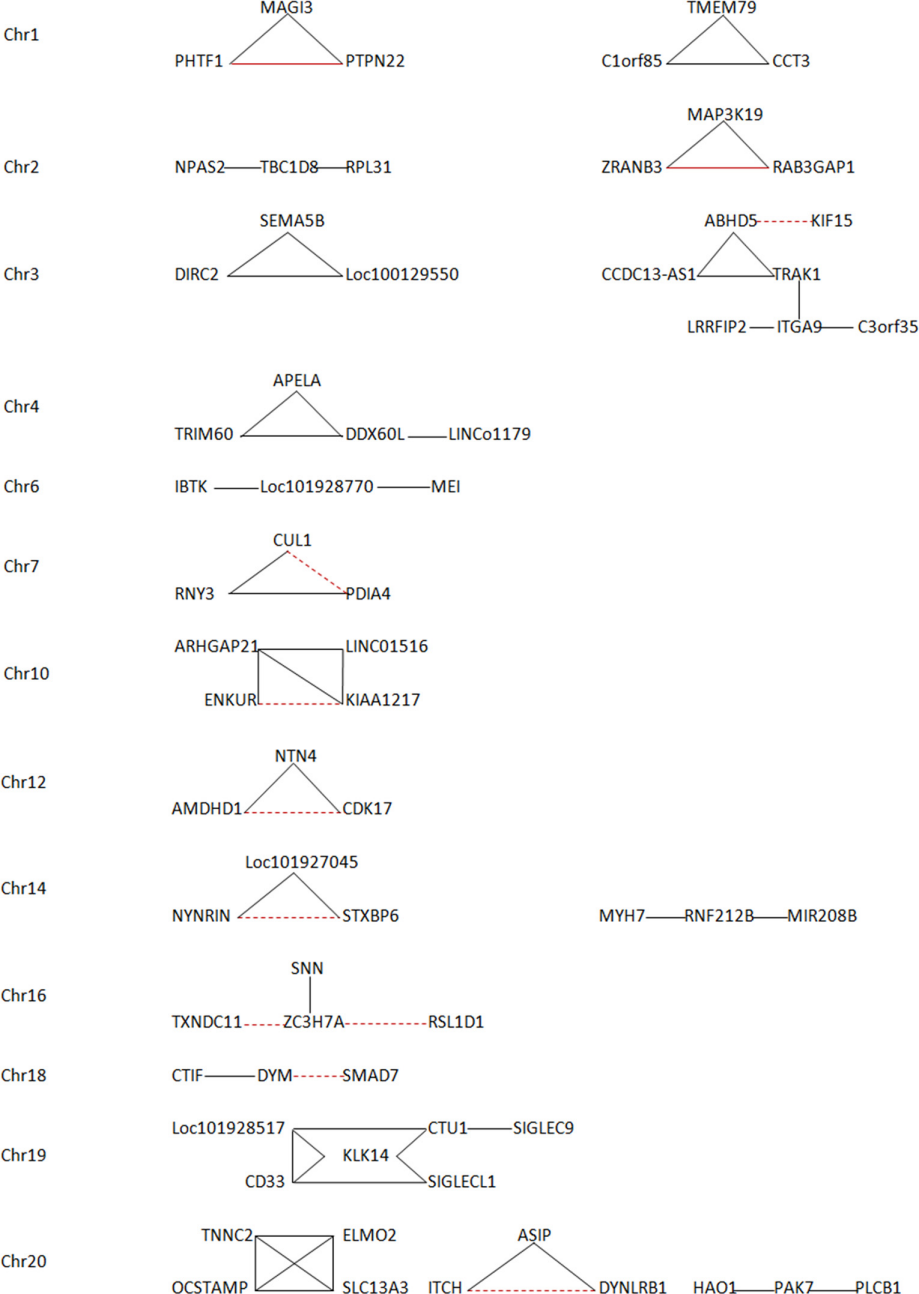
Nineteen networks of the interactions among three or more genes detected from the significant difference between the composite digenic and maximum zygotic LDs. The dashed red lines refer to the presence of a common interactor connecting two interacted genes from the information at http://thebiogrid.org/121402/summary/homo-sapiens/. The solid red lines refer to the presence of direct evidence from empirical studies.

Gene annotations indicate there are 751 SNP pairs (out of 2521) where each pair comes from the same annotated gene, 271 SNP pairs where each pair comes from two differently annotated genes, 909 SNP pairs where gene annotations are unknown, and 590 SNP pairs where one SNP comes from one annotated gene and the other from an unannotated gene in each pair. The feature is that these interactions occur between neighbor genes or regions (SNP pairs within 0.5 Mb). Some interactions have been evident in clinical experiments or via GWAS studies, while many more gene-gene interactions are not empirically corroborated yet. For instance, the interaction between BCL2L15 (rs7529353) and PHTF1 (rs1936398) on Chr1 (*d* = -0.0428±0.0032; *r* ^2^_*d<0*_ = 0.6324±0.5199) is reported to have protein-protein interactions coded by these two genes (http://www.bioinfo.mochsl.org.br/miriad/gene/BCL2L15/). Fig 6 lists 19 networks of the interactions among three or more genes on individual chromosomes. For instance, the interactions among genes MAGI3 (rs1230658), PHTF1(rs1936398) and PTPN22 (rs1217410) on Chr 1 are evident, which is associated with Type 1 diabetes [41, 42]. Part of ZRANB3 gene (213 bp; rs6742030) on Chr 2 is antisense to spliced RAB3GAP1 gene (rs7422031; *d* = -0.0483±0.0075; *r* ^2^_*d<0*_ = 0.6047±0.5590) according to the information from http://www.ncbi.nlm.nih.gov/IEB/Research/Acembly/. Many newly detected gene-gene interactions await experimental evidence (S3 Table).

## Discussion

In this study, we investigate the patterns of genome-wide zygotic LDs in eleven human populations and test the differences between the composite digenic and maximum zygotic LDs to search for epistatic SNPs. The results confirm the previous theory that patterns of zygotic LDs are more informative than that of the composite digenic/gametic LD in revealing natural population history [15, 21]. The eleven populations can be separated into a few groups in terms of the patterns of both zygotic and composite digenic LDs (Fig 4). Compared with the analysis of genome-wide gametic LD [22], zygotic LD analysis provides additional insights into the history of human populations. The four Africa populations (YRI, LWK, ASW, and MKK) have a fewer significant zygotic LDs than do the non-Africa populations in total. Africa populations contain more significant zygotic LDs for the double-homozygotes (*D*_*AABB*_) than for any other three zygotic LDs (*D*_*AABb*_, *D*_*AaBB*_, and *D*_*AaBb*_). The non-Africa populations, especially CEU, have more significant zygotic LDs, and contain more significant zygotic LDs for the double-heterozygotes (*D*_*AaBb*_) than for any other three zygotyic LDs (*D*_*AABB*_, *D*_*AABb*_, and *D*_*AaBB*_). Non-Africa populations also have many significant zygotic LDs for the double-homozygotes (*D*_*AABB*_), which is fewer than the number of significant *D*_*AaBb*_ but much more than the number of significant *D*_*AABb*_ or *D*_*AaBB*_. In addition, the four Africa populations have a fewer significant composite digenic LDs than do the non-Africa populations. All average r-squares (*r* ^2^_*AjBl*_ (*j* = *A*, *a*; *l* = *B*, *b*), *r* ^2^_*Δ*_, *r* ^2^_*d<0*_, and *r* ^2^_*d>0*_) increase in a general order of populations YRI, MKK, CEU, CHB, LWK, JPT, CHD,TSI, GIH, ASW, and MEX. Average r-squares are greater for *D*_*AABB*_ and *D*_*AaBb*_ than for *D*_*AaBB*_ and *D*_*AABb*_ in each population. Africa populations YRI and MKK can be separated from the other two Africa populations (LWK and ASW) in terms of the patterns of average r-squares. Patterns of r-squares with physical distances also vary among different zygotic LDs and among populations. All these divergences are essentially associated with their distinct population history.

The historical migration and origin of modern human are very complex from previous studies using different approaches (e.g., human anatomical structure and genetic approaches) and different types of molecular markers (e.g., mitochondrial DNA and Y-chromosome) [43, 44]. The current non-Africa populations are hypothesized to come out of Africa [45, 46] or arise from multiple regions [47, 48]. Under the Out of Africa model, the genetic variations in non-Africa populations reflect the historical divergence when tracing back to the precursor samples of original Africa ancestors. Because of the longer history, more extensive recombination could occur in situ modern Africa populations derived from ancient Africa populations, which results in lower LDs between SNPs in current Africa populations than in non-Africa populations [22, 49]. The results of zygotic LDs reflect this pattern. More significant zygotic LDs for both the double-homozygotes (*D*_*AABB*_) and double-heterozygotes (*D*_*AaBb*_) than for the homozygote-heterozygotes (*D*_*AABb*_ and *D*_*AaBB*_) are mainly due to the joint effects of selection and recombination (the maximum physical distance assayed here is 10 Mb). More significant zygotic LDs for the double-homozygotes occurring within a relative long distances on average (>1Mb, Table 3) suggest the main effects from genetic drift for the samples from original Africa ancestors. Genetic drift reduces the whole genome heterozygosity. Long physical distances enhance the occurrence of recombination events and hence indirectly amplify genetic drift effects [15], leading to over proportions of the double homozygotes. The more significant zygotic LDs for the double-heterozygotes occurring within shorter physical distances (<0.1Mb), on average, suggest that genotypic interactions from heterozygotes remain locally maintained on chromosomes. This could arise from the low recombination events within short physical distances, producing relatively smaller effects from the genetic drift. The original heterozygosities from ancestors could be relatively lightly eroded. Nature selection that enhances heterozygote interactions cannot be excluded. This also implies that ancestors harboured a pretty high level of genetic diversity due to the historical accumulation of mutation effects. The significant zygotic LDs for the heterozygote-homozygote represent an intermediate case. A certain level of recombination together with genetic drift effects can produce a fewer significant *D*_*AABb*_ ‘s or *D*_*AaBB*_’s.

The relatively fewer significant zygotic LDs in the group of ASW and LWK than in the group of YRI and MKK could reflect the difference in their genetic drift effects if all of them had approximately experienced the same length of evolutionary time. This is probably related to the divergence of different lines of descents in Africa, as implied from mitochondrial DNA markers (see review by Goldstein and Chikhi [44]). Our results imply that the effective population sizes (*N*_*e*_) could be smaller in populations ASW and LWK than in populations YRI and MKK.

Non-Africa populations CEU, CHB, CHD, JPT, and TSI exhibit the different patterns of zygotic LDs from those of the four Africa populations. The more total significant zygotic LDs are also consistent with their relatively younger populations that are reported to maintain higher gametic LDs [22, 44]. There are much more significant *D*_*AABB*_ and *D*_*AaBb*_ than significant *D*_*AaBB*_ and *D*_*AABb*_, reflecting the similar patterns to those in Africa populations. However, the pattern of more significant *D*_*AaBb*_ than significant *D*_*AABB*_ contrasts to the results in Africa populations. This is because *D*_*AABB*_ has greater fluctuations than *D*_*AaBb*_ when the genetic drift effects are large. These young populations, if derived from ancient Africa populations, could have small effective population sizes and larger genetic drift effects, under the Out of Africa model [45, 46] rather than the Multiregional model [47, 48]. Furthermore, genetic drift effects enhance the reduction in the difference between *D*_*AABB*_ and *D*_*AaBb*_ in magnitude. These joint effects produce more significant *D*_*AaBb*_ than significant *D*_*AABB*_ [15]. The differences among these populations mainly reflect historically different extents of their genetic drift effects.

It is of interest to discuss that the young CEU population has the most significant zygotic LDs (*D*_*AABB*_, *D*_*AaBB*_, *D*_*AABb*_, and *D*_*AaBb*_) among all eleven populations. One possible reason is that the genetic drift effects in CEU are smaller than other non-Africa populations, as implied from Tenesa et al.[49]. Tenesa and coworkers show that the effective population size is slightly larger in population CEU (*N*_*e*_ = 2772±598) than in populations JPT (2517±525) and CHB (2620±557) [49]. As a consequence, the zygotic LDs in population CEU have relatively smaller r-squares, closer to the patterns in Africa populations, and generally occur within shorter physical distances for *D*_*AABB*_, *D*_*AABb*_, and *D*_*AaBB*_ but within longer physical distances for *D*_*AaBb*_ in non-Africa populations.

Population MEX differs from other non-Africa populations in that it has more significant *D*_*AABB*_’s than significant *D*_*AaBb*_’s. It also differs from the Africa populations in that it has more significant zygotic LDs. One possible reason is that population MEX comes from an admixture of complex groups with potentially distinct ancestors [50]. This can be implied from the cluster analysis where population MEX has a probability of 78% to join two groups (CHD and JPT, GIH and TSI). Such case is analogous to the consequence of genomic admixture via gene flow, which maintains more extents of *D*_*AABB*_ than *D*_*AaBb*_ [15]. When the effects of gene flow (population admixture) are greater than or comparable to the genetic drift effects, it is expected the occurrence of more significant *D*_*AABB*_’s than significant *D*_*AaBb*_’s.

Our results provide an opportunity to evaluate a sole use of the composite digenic LD in revealing population history. In theory, the composite digenic LD is expected to have the pattern similar to that of gametic LD although it is always not smaller than the gametic LD in magnitude [25]. This trend becomes clearer when gametic LD (*D*_*AB*_) dominantly contributes to *Δ*_*AB*_ in most SNP pairs. It essentially reflects the information of low-order LD rather than higher-order LD [21, 51]. Our results confirm that the composite digenic LD generally does not have a stronger “spatial’ pattern along chromosomes than do any zygotic LDs (Table 4). Zygotic LDs decrease faster than the composite digenic LD with the physical distance, similar to the pattern of gametic LD [15]. It successfully indicates low LDs in populations YRI and MKK, but high LDs in populations ASW and LWK. This suggests that different ancestry populations might be historically involved in deriving the group of YRI and MKK and the group of ASW and LWK. The composite digenic LDs seem also to support that the admixture of distinct ancestry populations could be the main process for relatively higher LDs in population MEX, different from other non-Africa populations that have relatively lower LDs.

Apart from the utility of zygotic LDs for revealing population history, we search for genome-wide epistatic SNPs for fitness using zygotic LDs, which is now becoming an important issue in personal genetics [52]. This approach differs from the GWAS-based or other methods in that it does not require specific phenotypic traits, such as many complex diseases. Instead, the method works on a general fitness trait that is associated with different kinds of diseases directly or indirectly affecting human survivals. We have detected 19735–95921 SNP pairs that exhibit strong signals of epistatic selection (*d*<0, p-value<10^−9^). This accounts for a very small proportion of the total SNP pairs with significant zygotic LDs or significant composite digenic LDs. Nevertheless, variations among populations are substantial. The two Africa populations (ASW and LWK) have a few epistatic SNP pairs while CEU has the most expistatic SNP pairs. No common epistatic SNPs among all eleven populations suggest that these epistatic SNPs are mainly locally specific.

The feature of genome-wide epistatic SNPs is unknown since only a few epistatic SNPs in complex diseases are recorded in the literature [10, 27, 52]. Most reports refer to the major effects of individual SNPs in different disease traits via GWAS analysis. One common feature from our results is that genome-wide epistatic SNPs pairs generally tend to positively correlate with the physical distance, contrasting to the patterns of zygotic LDs in human populations. Further, epistatic SNPs generally occur within the physical distances that are, on average, longer than those for the composite digenic LDs but shorter than those for *D*_*AABB*_. Average r-squares for epistatic SNPs, *r* ^2^_*d<0*_, have similar patterns to other r-squares of zygotic LDs among populations (Table 2). This pattern helps to gain insights into the characteristics of genome-wide epistatic SNPs for fitness.

Another feature of genome-wide epistatic SNPs from our results is that many intron SNPs are involved in epistatic selection. The interacted intron SNPs are located within genes or between distinct genes (S1, S2 and S3 Tables). Also, gene annotations for many intron SNPs are unknown based on the latest online NCBI dbSNP in human genomes. This phenomenon is likely common in functional annotations in human or other organisms’ genomes [53], [54, 55]. More evidence that intron variants are associated with complex diseases would be rapidly accumulated in the literature with GWAS studies [56]. How these individual intron variants affect fitness remains complicated, such as altering transcriptions of disease-related genes (altering regulatory elements) [55] or changing the protein level of gene expressions. Elucidation of the molecular mechanisms and genetic pathways of these intron SNPs in forming epistasis remains an important challenge [3].

We have detected common epistatic SNP pairs in three regions (Africa, Europe, and East Asia) although no epistatic SNP pairs are shared among all eleven populations. This also suggests few common medications regarding specific SNP epistasis but the necessity of geographical medication [44]. Owing to the different numbers of populations among three regions investigated, the numbers of common epistatic SNPs are different (S1, S2 and S3 Tables). Gene annotations of all epistatic SNP pairs, including SNP reference sequence IDs and positions on individual chromosomes, are checked from current online NCBI dbSNP. The unannotated SNPs in S1, S2 and S3 Tables could be annotated with the accumulation of future functional gene mapping. The feature for regionally common epistatic SNPs is that they occur within less than 1Mb, including SNPs within genes and between neighbour genes. Some epistatic SNPs are evident from clinical trials or the experiment reports in the literature, but many more epistatic SNP pairs are not verified or newly discovered. These newly found epistases await empirical evidence. All these epistatic SNP pairs are of potential importance for understanding common diseases-common variants (CD-CV) in different regions and for aiding in geographical genomic medicine [52].

## Supporting Information

S1 Table Gene annotations of the common SNP pairs among four Africa populations (ASW, LWK, MKK, and YRI), with the composite digenic LD being significantly smaller than the maximum zygotic LD (p-value<10^−9^). (XLSX)

S2 Table Gene annotations of the common SNP pairs among three Asia populations (CHB, CHD, and JPT), with the composite digenic LD being significantly smaller than the maximum zygotic LD (p-value<10^−9^). (XLSX)

S3 Table Gene annotations of the common SNP pairs between two Europe populations (CEU and TSI), with the composite digenic LD being significantly smaller than the maximum zygotic LD (p-value<10^−9^). (XLSX)
